# Impairments in personality functioning in adolescents with anorexia nervosa

**DOI:** 10.1002/erv.3146

**Published:** 2024-10-28

**Authors:** Andrea M. Schumacher, Armita Tschitsaz, Stefan Lerch, Andrea Wyssen, Franziska Schlensog‐Schuster, Ines Mürner‐Lavanchy, Julian Koenig, Marialuisa Cavelti, Michael Kaess

**Affiliations:** ^1^ University Hospital of Child and Adolescent Psychiatry and Psychotherapy University of Bern Bern Switzerland; ^2^ Division of Youth Mental Health Faculty of Psychology University of Basel Basel Switzerland; ^3^ Department of Child and Adolescent Psychiatry Psychosomatics and Psychotherapy Faculty of Medicine and University Hospital Cologne University of Cologne Cologne Germany; ^4^ Department of Child and Adolescent Psychiatry Centre for Psychosocial Medicine University of Heidelberg Heidelberg Germany

**Keywords:** adolescence, alternative model of personality disorder, eating disorder, personality disorder, personality functioning

## Abstract

**Objective:**

Impairments in personality functioning (PF) according to the Alternative Model of Personality Disorders (AMPD) may affect anorexia nervosa (AN) and its treatment. However, PF impairments in adolescents with AN have not been studied. This case‐control study analysed PF impairments in adolescent inpatients suffering from AN compared to a clinical control group (CC) and investigated the relationship of PF with AN severity.

**Method:**

Adolescent patients with AN (*n* = 43) and CC (*n* = 127) were matched by age, sex, and global functioning. Validated interviews were applied to assess impairments in PF (Semi‐Structured Interview for Personality Functioning, STiP‐5.1) and severity of AN (Eating Disorder Examination, EDE), as well as weight measures. Group comparisons of PF impairment and the relationship between PF impairment and AN severity were analysed using multiple linear regression.

**Results:**

AN patients showed greater impairment in the facet ‘*self‐esteem*’ (*p* = 0.033, Cohen's *f* ^*2*^ = 0.036) than CC. In AN patients, impairments in the domain ‘*self‐functioning’* (*p* = 0.003, Cohen's *f* ^*2*^ = 0.254) and its underlying element ‘*identity*’ (*p* = 0.016, Cohen's *f* ^*2*^ = 0.172) were associated with AN severity (EDE). Additionally, higher impairments in two facets of ‘*empathy*’ (*p* = 0.019, Cohen's *f* ^*2*^ = 0.223) and ‘*intimacy*’ (*p* = 0.026, Cohen's *f* ^*2*^ = 0.199) were associated with higher AN severity (lower BMI percentiles).

**Conclusions:**

These results support theoretical frameworks of AN and highlight the importance of PF impairments in understanding and treating AN. Future research should investigate how impairments in PF affect AN development and treatment response through longitudinal studies.

## Highlights

- Adolescents with anorexia nervosa, compared with clinical controls, showed significant deficits in the personality functioning facet ‘*self‐esteem’.*
- Deficits in ‘*self‐functioning*’, and particularly ‘*identity’*, were moderately associated with greater anorexia nervosa symptoms (EDE), and deficits in ‘*interpersonal functioning*’ were moderately associated with lower BMI percentiles.
- When treating adolescents with anorexia nervosa, deficits in ‘*self‐functioning*’ and ‘*interpersonal functioning’* should be addressed.

## 1 INTRODUCTION

Anorexia nervosa (AN) is a severe mental illness that is associated with an increased mortality rate, despite inpatient treatment (Van Eeden et al., 2021; Van Hoeken & Hoek, 2020) and represents a significant global burden (Wu et al., 2020). It is therefore crucial to understand the factors that affect its development, maintenance and treatment. The cognitive interpersonal model of AN by Treasure et al. (2020) highlights the complex interplay of biological, psychological, psychosocial and behavioural factors that contribute to the development and maintenance of the disorder. It also emphasises the influence of personality pathology as a potential vulnerability factor, consistent with previous studies reporting that certain personality disorders (PDs) and personality traits are common in individuals with AN.

Regarding PDs, restrictive AN was associated with comorbid obsessive‐compulsive, avoidant and dependent PDs, while binge‐purging AN in particular exhibited additional associations with borderline and paranoid PDs (Farstad et al., 2016). This study used a mixed sample of adults and adolescents and was based on the categorical model of personality pathology according to the Diagnostic and Statistical Manual of Mental Disorders (DSM‐IV; American Psychiatric Association, 1994) and the International Statistical Classification of Diseases and Related Health Problems (ICD‐10; World Health Organization, 2016). Meta‐analytic research has shown elevated levels of ‘perfectionism’ in individuals with AN compared to both clinical controls (CC) and healthy controls (Dahlenburg et al., 2019). Adolescents diagnosed with eating disorders (EDs) have shown enhanced levels of personality traits associated with negative affectivity, detachment, and conscientiousness compared to non‐ED adolescents, with conscientiousness traits more strongly related to AN than to other EDs (Dufresne et al., 2020).

However, the categorical approach used to assess PD has widely documented shortcomings: frequent co‐occurrence of multiple PD diagnoses (Morey et al., 2011), insufficient evidence for the 10 individual PD diagnoses, unclear distinction between normal and pathological psychological functioning, arbitrary diagnostic thresholds, substantial heterogeneity within individual PDs, high proportion of individuals who do not meet a specific PD but ‘other specified’ or ‘unspecified PD’, and limited clinical utility (Morey et al., 2015; Widiger et al., 2009; Zimmermann et al., 2015). As a result, the model lacks empirical support due to low reliability and external validity (for an overview see Hopwood et al., 2018). In recognition of these drawbacks, and with respect to research highlighting the importance of including subsyndromal personality impairment in AN (Rohde et al., 2023), one strategy to address these challenges is to conceptualise personality on a dimensional continuum. This paradigm shift, exemplified by the Alternative Model for Personality Disorders (AMPD; American Psychiatric Association, 2013), moves away from viewing personality in terms of discrete entities. Instead, it is thought to provide a more comprehensive understanding of its complexity and may allow a more extensive examination of the relationship between AN and personality. The AMPD defines PD in terms of impairments in personality functioning (PF, criterion A). PF is assessed by means of self‐ and interpersonal functioning, covering impairments in identity, self‐direction, intimacy and empathy. The Level of Personality Functioning Scale (LPFS; American Psychiatric Association, 2013) is used to rate the degree of impairment, and assessing criterion A by means of a semi‐structured interview is recommended. Criterion B of the AMPD encompasses maladaptive personality traits. Thus, the AMPD allows the detection of a general PD, as well as combined and subsyndromal personality pathology described by means of PF and pathological personality traits.

The introduction of the AMPD, and in particular, criterion A, raises the question of whether individuals with AN exhibit specific patterns of impairment in PF and whether there is a link between PF and AN severity. Biberdzic et al. (2021) examined PF in a student sample (aged 18–25 years) with disordered eating behaviours. Results showed that individuals with eating problems had greater impairment in the self‐ and interpersonal domains of PF according to the LPFS with small to medium effect sizes (Biberdzic et al., 2021). Klein et al. (2022) found that impaired PF was correlated with increased ED symptoms *(r* = 0.22; *p* < 0.01) in a community‐based sample (aged 14–92 years). In a clinical sample (aged 18–64 years) including restrictive and binge‐purging types of AN and patients with bulimia nervosa, a weak association was found between greater impairment in PF and ED pathology (Rohde et al., 2023). However, the latter two studies measured impairment in PF using self‐report instruments.

To our knowledge, no study to date has examined impairments in PF specifically in adolescents with AN using a semi‐structured clinical interview that assesses all aspects of PF (including impairments in identity, self‐direction, intimacy and empathy) according to AMPD Criterion A. However, a few studies have assessed PF in clinical adolescent samples using semi‐structured interviews. For instance, adolescents with and without personality disorders exhibited moderate to severe PF impairment (Weekers et al., 2021), while a mixed sample of outpatient and inpatient adolescents showed impairment ranging from adaptive functioning to moderate impairment across PF aspects (Wyssen et al., 2024).

As AN has its high‐risk period in adolescence (Nagl et al., 2016), it may be particularly important to study impairments in PF in clinical populations with AN in this age group, as the ED pathology is often not yet chronic. Therefore, it may be crucial to understand and identify deficits in PF during this period and use it as a potential treatment target.

To address these gaps, we used a semi‐structured clinical interview to investigate: (a) whether adolescent inpatients with AN have similar PF characteristics compared to a general clinical psychiatric sample without AN diagnosis. Due to the limited research, an exploratory, non‐hypothesis‐driven approach was adopted for this comparison. Furthermore, we examined (b) whether impairments in PF are correlated with AN severity within the group of adolescent inpatients with AN. Based on the existing literature, we expected a positive correlation between impairment in PF and AN severity.

## 2 METHOD

### 2.1 Participants and procedures

**FIGURE 1:**
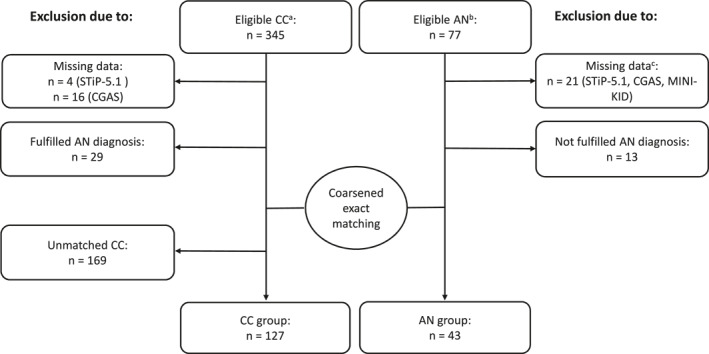
Flow chart of the inclusion and exclusion of participants for AN and CC groups. STiP‐5.1, Semi‐structured Interview for Personality Functioning *DSM‐5;* CGAS, Children's Global Assessment Scale; MINI‐KID, Mini International Neuropsychiatric Interview for Children and Adolescents. ^a^Eligible CC group: includes participants with data exclusively from the BeBaDoc study. ^b^Eligible AN group: includes 56 participants with data in the Anorexia Registry Study and in the BeBaDoc study and a further 21 participants with data only in the Anorexia Registry Study. ^c^21 participants with data only in the Anorexia Registry Study were excluded because they had no data (e.g., STiP‐5.1, CGAS, MINI‐KID) in the BeBaDoc study.

Data for the current study were pooled from two studies: the Anorexia Registry Study (Herpertz‐Dahlmann & Hebebrand, 2017), which started in July 2021 and is ongoing, and the Bern Basic Documentation, BeBaDoc, which was conducted between November 2018 and December 2022. This analysis includes data collected from both studies until October 2022. Participants were recruited from general psychiatric inpatient and day‐care units, as well as inpatient‐equivalent home treatment (BeBaDoc study) and a specialised therapy centre for EDs (Anorexia Registry Study) at the University Hospital of Child and Adolescent Psychiatry and Psychotherapy Bern, Switzerland. A detailed overview is provided in the flow chart (Figure 1).

Inclusion criteria for the BeBaDoc study were an age of 11–18 years and sufficient German language skills. Exclusion criteria were problems in understanding study details or giving informed consent. The inclusion criteria for the Anorexia Registry Study were identical to those of the BeBaDoc study, with the additional requirement of a diagnosis of AN based on clinical judgement (ICD‐10: F50.00, F50.01; F50.1; World Health Organization, 2016). Participants in the CC group of the current study were selected from the BeBaDoc study if they did not meet the diagnostic criteria for AN according to the Mini International Neuropsychiatric Interview for Children and Adolescents (MINI‐KID). Participants in the AN group were selected from the Anorexia Registry Study if they met the MINI‐KID diagnostic criteria for AN. Written informed consent was obtained from all participants, and also from parents for those under 14 years of age. After admission to the clinic, assessments were carried out by well‐trained PhD students or undergraduate psychology students. The studies were conducted in accordance with the Declaration of Helsinki (World Medical Association, 2013) and were approved by the local ethics committees (BeBaDoc Ethics ID: 2018‐01339; and Anorexia Registry Ethics ID: 2021‐00234).

### 2.2 Measures

*Demographic Data.* Demographic information was collected using a standardised set of questions to assess age, sex and type of school.

*Assessments of the BeBaDoc study (completed by both samples AN and CC samples)*

*The Mini International Neuropsychiatric Interview for Children and Adolescents (MINI‐KID).* To assess current major and comorbid psychiatric disorders according to DSM‐IV and ICD‐10, the MINI‐KID, (Sheehan et al., 2010), was used. It is a structured interview that allows for valid and reliable psychiatric diagnoses for children and adolescents (Duncan et al., 2018).

*Children's Global Assessment Scale (CGAS).* Global level of functioning was assessed by the CGAS (Shaffer et al., 1983), a single‐item instrument designed to assess psychological and social functioning on a scale of 1 to 100, with low scores indicating high functional impairment and high scores indicating low impairment. A score above 70 is considered as a threshold for normal functioning. Good psychometric properties have been demonstrated (Shaffer et al., 1983).

*The Semi‐Structured Interview for Personality Functioning (STiP‐5.1).* The STiP‐5.1 was used for the AMPD criterion A, which measures the ‘core of PD’ by assessing the severity of personality impairment (Hutsebaut et al., 2017). The level of PF is evaluated for the domains of self‐ and interpersonal functioning, defined by four elements (identity, self‐direction, empathy and intimacy), each with three facets (a total of 12; see eTable 1). For each of the 12 facets (e.g., impact or self‐esteem), the degree of functioning can be determined by five levels according to the LPFS: low to no impairment (0), mild (1), moderate (2), severe (3) or extreme (4) impairment, with higher scores indicating a higher degree of personality impairment. The total score includes the average of all elements. The domains are calculated as the average of their respective elements, consisting of identity and self‐direction for the self‐domain and empathy and intimacy for the interpersonal domain. The elements are based on averaging the three corresponding facets, and the facets correspond to the raw scores. In addition, a score of two (moderate impairment) or higher in at least two elements was used as the diagnostic threshold for a PD diagnosis according to the DSM‐5 AMPD guidelines for trait specified and disorder specified PD. Good psychometric properties in adults and adolescents have been reported (Hutsebaut et al., 2017; Weekers et al., 2021; Zettl et al., 2019). Internal consistency (Cronbach's *α*) of the total score in the present study was α = 0.87, of the self‐functioning α = 0.83 and interpersonal functioning domain α = 0.82, and ranged from 0.69 to 0.79 for the four elements.

*Assessments of the Anorexia Registry Study (completed only by the AN sample)*

*Eating Disorder Examination (EDE).* The German version of the semi‐structured interview EDE (Hilbert et al., 2004), which captures cognitive‐emotional and behavioural characteristics, was used to assess the AN severity. The EDE comprises 28 items, 22 of which can be divided into four subscales (restraint eating, eating concern, shape concern, weight concern). Participants rated the frequency of the experiences over the past 28 days on a scale from 0 = item was not fulfilled to 6 = item was fulfiled every day or to an extreme degree for 14 of these items. Additionally, intensity ratings ranging from 0 = characteristic was not present to 6 = characteristic was present to an extreme degree were assigned to eight items. At the same time, 14 of the 28 items can be used to define ED diagnoses according to DSM‐IV. The global score, defined as the mean of the four subscales, reflecting the global extent of current ED pathology, was used in the current analysis. The EDE has been shown to have good psychometric properties in young adults (Hilbert et al., 2004). The internal consistency (Cronbach's *α*) of the global score was 0.88.

*BMI percentiles and z‐scores.* An additional indicator of AN severity are the age‐ and sex‐adjusted body mass index percentiles (BMI percentiles), based on the World Health Organization (WHO) growth standards and the Centres for Disease Control and Prevention (CDC; Kuczmarski et al., 2000). BMI percentiles are recommended when assessing weight in children and adolescents, as they take into account developmental norms and sex at different ages (American Psychiatric Association, 2013). Additionally, we used the corresponding *z*‐scores from the BMI percentiles, as percentiles have limited utility when applied to cohorts that include participants with extreme BMI values (e.g., below the first percentile; Wang & Chen, 2012). In a normal distribution, a *z*‐score indicates the distance in standard deviations (*SDs*) from a specific value to the average value of the distribution, with a *z*‐score of 0 representing an average score, and a *z*‐score of +1/‐1 meaning the score is one *SD* above/under population average (Martinez‐Millana et al., 2018).

### 2.3 Statistical analyses

Patients of the AN and CC groups were matched regarding age, sex and global functioning (CGAS) using Coarsened Exact Matching (CEM), with calculated weights for each stratum where the global functioning is coarsened to 1–10, 11–20, …, 91–100. CEM allows for weighted matching and is often used when the CC group is larger than the targeted group (i.e., AN; Iacus et al., 2008). This means that if the matching criteria were met, it was possible for each patient with AN to be matched with more than one CC patient. For descriptive purposes, means and *SDs* were calculated for the continuous variables, and exact numbers and proportions for the nominal variables.

To calculate potential differences between AN and CC patients, linear weighted regression analyses were performed with group (AN vs. CC) as the independent variable and PF scores as the dependent variable. Each aspect of PF (i.e., domains, elements, facets) was individually tested, resulting in a total of 19 models. Age and global functioning were included as control variables to increase accuracy, as potential confounding effects of these variables may persist after matching and can be minimised by including them in the analysis (see Pearce, 2016). To address multiple testing, the Max‐T method with 10,000 repetitions (Collins Dekker; ter Braak, 2022) was applied for *p*‐value correction. For all regression analyses, the residual distribution was visually checked, showing no multi‐collinearity (VIF <5), and no heteroscedasticity for group (non‐significant Breusch‐Pagan test).

To investigate whether PF is associated with AN severity, a series of multiple linear regression analyses were performed with the EDE global score, BMI percentiles and BMI percentile *z*‐scores as outcome variables. Percentiles <1 were treated as zero. Four separate models were fitted for each outcome variable, including (1) the STiP‐5.1 total score, (2) the domains, (3) the elements, and (4) the facets as predictors, resulting in a total of 12 models. Global functioning was included in all models as control variable to account for its potential influence on the relationship. Potential outliers were identified by examining standardised residuals equal to or exceeding plus or minus three (Osborne & Overbay, 2004). In addition, in univariate analyses, we calculated the Pearson correlation coefficients between each STiP‐5.1 score and AN severity indicator separately and *p*‐values were corrected using the Max‐T method with 10,000 repetitions. Cohen's *f* ^*2*^ effect size was calculated for all linear regression analyses, with a Cohen's *f* ^*2*^ of 0.10 being interpreted as a small effect, 0.25 as a medium effect and 0.50 as a large effect (Cohen, 1988). All *p*‐values were two‐sided and the alpha level was set at 0.05. Statistical analyses were performed using STATA statistical software version 17 (StataCorp, 2021).

## 3 RESULTS

### 3.1 Participants

Combining data from the two studies (BeBaDoc, *n* = 401, and the Anorexia Registry Study, *n* = 77) resulted in 345 participants with data from the BeBaDoc study only, 56 with data from both studies, and a further 21 participants with data from the Anorexia Registry Study only who were excluded due to missing data from the MINI‐KID, CGAS, and STiP‐5.1. Of the 345 participants with data from the BeBaDoc study only, 29 participants were excluded because they met the MINI‐KID diagnostic criteria for AN (i.e., an exclusion criterion for the CC group) and a further 20 participants were excluded because of missing STiP‐5.1 (*n* = 4) and CGAS (*n* = 16) data. 13 of the 56 participants with data from both studies were excluded, because they did not fulfil the criteria for AN as assessed by the MINI‐KID (i.e., an inclusion criterion for the AN group). After conducting the CEM (resulting in 169 unmatched CC participants), a final sample of *N* = 170 remained, including 127 CC, who were matched to 43 patients with AN. A detailed overview is provided in the flow chart (Figure 1).

The mean age of the sample was 14.88 years (*SD* = 1.04; range 12−16 years) and all participants were female. Of the 43 patients with AN, 30.95% fulfilled at least one comorbid psychiatric diagnosis, reported an illness duration of 14.5 months, had an EDE global score of 3.86 (*SD* = 1.05) and BMI percentiles ranging from <1 to 36 (corresponding *z*‐scores ranged from −5.76 to −0.35). 13 (30.23%) patients with AN and 46 (36.22%) of the CC patients met the diagnostic PD threshold (≥2 LPFS on two or more elements) according to the AMPD. The majority (76%–85%) of the CC patients suffered from the co‐occurrence of multiple psychiatric diagnoses such as affective or anxiety disorders. Demographic and clinical descriptive statistics for the total sample and for each group separately are presented in Table 1 and eTable 3.

**TABLE 1:** Characteristics of the total sample and by clinical status (AN and CC).

Characteristics	Total sample (*n* = 170)	An (*n* = 43)	CC (*n* = 127)
Age (mean, *SD)*	14.88, 1.04	14.60, 1.03	14.97, 1.04
Global level of functioning (CGAS; mean, *SD*)	49.79, 11.58	51.60, 13.05	49.17, 11.03
Highest level of education (*n*, %)^a^			
Primary school (ISCED level 0−1; at least 6 school years)	20, 11.76	3, 6.98	17, 13.38
Secondary school (ISCED level 2; 9−10 school years)	115, 67.65	30, 69.77	85, 66.93
High school (ISCED level 3; 12−13 school years)	35, 20.59	10, 23.26	25, 19.69
Diagnostic threshold for PD according to the AMPD (STiP‐5.1; *n*, %)	59, 34.71	13, 30.23	46, 36.22
Eating disorder diagnoses (MINI‐KID; *n*, %)			
Bulimia nervosa	16, 9.41	‐	16, 12.60
Anorexia nervosa diagnoses			
AN restrictive type	42, 24.71	42, 97.67	‐
AN binge‐purging type	1, 0.59	1, 2.33	‐
Other (comorbid) psychiatric diagnoses (MINI‐KID; *n*, %)			
Any affective disorder	130, 76.47	33, 76.74	97, 76.38
Any anxiety disorder	133, 78.24	25, 58.14	108, 85.04
Any obsessive‐compulsive disorder	40, 23.53	7, 16.28	33, 25.98
Any posttraumatic stress disorder	33, 19.41	5, 11.63	28, 22.05
Any substance use disorder	43, 25.29	1, 2.33	42, 33.07
Any behavioural and emotional disorders	40, 23.53	2, 4.65	38, 29.92
Any psychotic disorder	34, 20.00	7, 16.28	27, 21.26
Duration of AN in months (mean, *SD*)	‐	14.51, 13.39	‐
EDE global score (mean, *SD*)	‐	3.86, 1.05	‐
BMI percentiles (mean, *SD*)	‐	3.09, 6.17	‐
BMI percentiles *z*‐score (mean, *SD*)	‐	−2.79, 1.33	‐

Abbreviations: AN, Anorexia nervosa group; BMI, Body mass index; CC, Clinical control group; CGAS, Children's Global Assessment Scale; EDE, Eating Disorder Examination; ISCED, International Standard Classification of Education; STiP‐5.1, Semi‐structured Interview for Personality Functioning *Diagnostic and Statistical Manual of Mental Disorders, Fifth Edition*; MINI‐KID, Mini International Neuropsychiatric Interview for Children and Adolescents.

^a^ Education levels are based on International Standard Classification of Education (ISCED).

### 3.2 Group differences of PF in AN and CC patients

Weighted regression analyses revealed that the group variable (AN vs. CC; predictor) did not explain the variance in the outcome variable STiP‐5.1 total score, nor in the domains or elements (see Table 2 and eTable 4 in the supporting information). However, on the facet level, more impairment in *‘self‐esteem’*, which is part of the element ‘*identity*’, was found in patients with AN compared to CC (*b* = 0.408, corrected *p*‐value = 0.033; model: F(3,166) = 23.63, *p* < 0.001, *n* = 170), with a Cohen's *f* ^*2*^ effect size of 0.036. Additionally, patients with AN tended to be less impaired in ‘*impact*’, which is part of the element ‘*empathy*’, than CC (*b* = −0.369, corrected *p*‐value = 0.039; model: F(3,166) = 6.98, *p* < 0.001, *n* = 170), with a Cohen's *f* ^*2*^ effect size of 0.028. No significant group differences were found for the other facets (see Table 2). Both reported differences at the facet level remained robust and did not change when a sensitivity analysis was performed in which individuals with a diagnosis of bulimia nervosa were excluded from the CC group (see eTable 6).

**TABLE 2:** Group differences in PF between AN and CC patients.

STiP‐5.1	Group differences *B*	*SE B*	95% CI	*p*	*p adj*.	*f* ^*2*^
			*LL UL*			
Total score	−0.073	0.113	−0.295 0.150	0.520	0.557	0.003
Domain						
Self	−0.033	0.135	−0.299 –0.234	0.809	0.827	0.000
Interpersonal	−0.113	0.120	−0.349 0.124	0.349	0.367	0.005
Element						
Identity	−0.013	0.144	−0.298 0.271	0.927	0.934	0.000
Self‐direction	−0.052	0.157	−0.362 0.258	0.740	0.769	0.001
Empathy	−0.234	0.124	−0.478 0.010	0.061	0.059	0.022
Intimacy	0.009	0.150	−0.288 0.306	0.955	0.963	0.000
Facet						
Identity						
Experience of oneself as unique	−0.106	0.196	−0.494 0.281	0.588	0.634	0.002
Self‐esteem	0.408	0.167	0.079 0.737	0.016*	0.033*	0.036
Emotions	−0.341	0.186	−0.709 0.026	0.069	0.106	0.020
Self‐direction						
Goals	−0.123	0.198	−0.515 0.269	0.536	0.580	0.002
Norms^a^	0.104	0.228	−0.346 0.553	0.649	0.696	0.001
Self‐reflection	−0.141	0.194	−0.525 0.243	0.470	0.529	0.003
Empathy						
Understanding others^a^	−0.139	0.163	−0.460 0.182	0.394	0.404	0.004
Perspectives	−0.197	0.152	−0.497 0.104	0.198	0.212	0.010
Impact	−0.369	0.172	−0.709 –0.030	0.033*	0.039*	0.028
Intimacy						
Connection	−0.022	0.208	−0.433 0.389	0.915	0.925	0.000
Closeness	0.073	0.182	−0.286 0 0.431	0.690	0.728	0.001
Mutuality	−0.025	0.157	−0.335 0.286	0.876	0.877	0.000

*Note*: *n* = 170.

Abbreviations: *B,* unstandardised regression coefficient; *f* ^*2*^ *,* Cohen's *f*; LL, lower level; *p* adj., adjusted *p*‐value; *SE B*, standard error of *B*; STiP‐5.1, Semi‐structured Interview for personality functioning *Diagnostic and Statistical Manual of Mental Disorders, Fifth Edition*; UL, upper level.

^a^ One missing value, *n* = 169.

*p < 0.05.

### 3.3 Relationship between PF and AN severity

#### 3.3.1 EDE global score

A series of multiple linear regression analyses revealed that higher impairments in the STiP‐5.1 total score (*b* = 0.520, *p* = 0.034, Cohen's *f* ^*2*^ = 0.120; model: F(2,40) = 2.56, *p* = 0.090, *n* = 43) and the domain ‘*self‐functioning’* (*b* = 0.712; *p* = 0.003, Cohen's *f* ^*2*^ = 0.254; model: F(3, 39) = 3.65, *p* = 0.021, *n* = 43) were associated with a higher EDE global score. Additionally, at the element level, higher impairment in *‘identity’* was associated with a higher EDE global score (*b* = 0.689, *p* = 0.016, Cohen's *f* ^*2*^ = 0.172; model: F(5,37) = 2.57, *p* = 0.043, *n* = 43). None of the other STiP‐5.1 domains, elements or facets were associated with the EDE global score (Table 3).

**TABLE 3:** Results of multiple regression analyses in patients with AN: Prediction of AN severity by PF.

STiP‐5.1	EDE global *B*	*SE B*	95% CI	*p*	*f* ^*2*^	BMI P *B* ^a^	*SE B* ^a^	95% CI^a^	*p* ^a^	*f* ^*2*^ ^a^	BMI *z* *B*	*SE B*	95% CI	*p*	*f* ^*2*^
			*LL UL*					*LL UL*					*LL UL*		
Total score	0.520	0.237	0.041 0.998	0.034*	0.120	0.576	0.807	−1.056 2.208	0.480	0.013	0.201	0.312	−0.429 0.830	0.523	0.010
Domain															
Self	0.712	0.226	0.254 1.169	0.003*	0.254	0.796	0.825	−0.874 2.465	0.341	0.025	0.385	0.313	−0.248 1.017	0.226	0.039
Interpersonal	−0.276	0.258	−0.798 0.247	0.293	0.029	−0.307	0.933	−2.196 1.582	0.744	0.003	−0.237	0.357	−0.960 0.486	0.511	0.011
Element															
Identity	0.689	0.273	0.135 1.243	0.016*	0.172	0.573	0.997	−1.449 2.595	0.569	0.009	0.719	0.374	−0.039 1.478	0.062	0.100
Self‐direction	0.001	0.279	−0.565 0.567	0.997	0.000	0.658	1.014	−1.398 2.713	0.521	0.012	−0.264	0.383	−1.040 0.511	0.494	0.013
Empathy	−0.029	0.294	−0.624 0.566	0.923	0.000	0.969	1.088	−1.238 3.175	0.379	0.022	0.346	0.402	−0.470 1.161	0.396	0.020
Intimacy	−0.136	0.265	−0.674 0.401	0.610	0.007	−1.268	1.006	−3.309 0.773	0.216	0.044	−0.410	0.363	−1.146 0.326	0.266	0.034
Facet															
Identity															
Experience of oneself as unique	0.248	0.221	−0.204 0.700	0.271	0.043	1.086	0.649	−0.243 2.416	0.105	0.100	0.173	0.305	−0.451 0.797	0.574	0.011
Self‐esteem	−0.282	0.288	−0.870 0.307	0.336	0.033	0.463	0.842	−1.262 2.188	0.587	0.011	0.466	0.398	−0.347 1.280	0.251	0.047
Emotions	0.433	0.211	0.001 0.865	0.050	0.145	0.304	0.617	−0.960 1.568	0.626	0.009	0.256	0.292	−0.341 0.853	0.388	0.026
Self‐direction															
Goals	0.051	0.245	−0.449 0.551	0.837	0.001	1.180	0.776	−0.410 2.771	0.140	0.083	0.369	0.338	−0.322 1.060	0.284	0.041
Norms	0.064	0.217	−0.379 0.507	0.769	0.003	0.053	0.647	−1.273 1.379	0.936	0.000	−0.333	0.299	−0.946 0.279	0.275	0.043
Self‐reflection	0.280	0.224	−0.179 0.739	0.222	0.054	−1.247	0.662	−2.603 0.108	0.070	0.127	−0.431	0.310	−1.065 0.203	0.175	0.067
Empathy															
Understanding others	0.035	0.224	−0.422 0.493	0.876	0.001	−1.636	0.654	−2.976 –0.296	0.019*	0.223	−0.352	0.309	−0.983 0.280	0.264	0.045
Perspectives	0.273	0.324	−0.389 0.936	0.405	0.025	1.261	0.946	−0.676 3.198	0.193	0.064	−0.094	0.447	−1.009 0.821	0.835	0.002
Impact	0.020	0.246	−0.482 0.522	0.935	0.000	1.476	0.721	−0.001 2.953	0.050	0.150	0.446	0.339	−0.248 1.140	0.199	0.059
Intimacy															
Connection	−0.081	0.227	−0.547 0.384	0.723	0.004	−1.573	0.667	−2.938 –0.207	0.026*	0.199	−0.097	0.314	−0.739 0.546	0.760	0.003
Closeness	−0.334	0.256	−0.858 0.190	0.203	0.059	0.014	0.753	−1.528 1.557	0.985	0.000	−0.090	0.354	−0.814 0.633	0.800	0.002
Mutuality	−0.175	0.261	−0.709 0.359	0.509	0.015	−0.065	0.795	−1.694 1.564	0.936	0.000	−0.018	0.361	−0.756 0.721	0.961	0.000

*Note*: *n* = 43.

Abbreviations: *B,* unstandardised regression coefficient; BMI P, Body mass index percentiles; BMI z, Body mass index percentile z‐scores; EDE global, eating disorder examination global score; *f* ^*2*^ *,* Cohen's *f*; LL, lower level; *p* adj., adjusted *p*‐value; *SE B*, standard error of *B*; STiP‐5.1, Semi‐structured Interview for personality functioning *Diagnostic and Statistical Manual of Mental Disorders, Fifth Edition*; UL, upper level.

^a^ BMI *p* values based on *n* = 42 as one outlier was removed.

*p < 0.05.

### 3.4 BMI percentiles

During the analysis, an outlier with a standardised residual of 4.60 was identified. To ensure the robustness of the results, this outlier was removed from the data set. After excluding this outlier, a series of multiple linear regression analyses revealed that at the facet level, higher impairment in ‘*understanding others*’, part of the ‘*empathy*’ element, (*b* = −1.636, *p* = 0.019, Cohen's *f* ^*2*^ = 0.223; model: F(13,28) = 2.33, *p* = 0.030, *n* = 42; see Table 3) and higher impairment in ‘*connection*’, part of the ‘*intimacy*’ element, was associated (*b* = −1.573, *p* = 0.026, Cohen's *f* ^*2*^ = 0.199; model: F(13,28) = 2.33, *p* = 0.030, *n* = 42; see Table 3) with lower BMI percentiles. None of the other STiP‐5.1 total, domain, or element scores were associated with BMI percentiles (Table 3).

### 3.5 BMI percentile *z*‐scores

None of the STiP‐5.1 scores sign predicted BMI percentile *z*‐scores significantly (Table 3).

eTable 5 shows the overall model fit of all 12 models of AN severity indicators. Additionally, the results of the univariate analyses (Pearson correlation) between PF and AN severity indicators are presented in eTable 2.

## 4 DISCUSSION

The aim of the present cross‐sectional study was to examine impairments in PF according to the AMPD by comparing adolescent inpatients suffering from AN to a CC group. In addition, the relationship between impairments in PF and AN severity was investigated in AN patients.

Three main results emerged: First, AN patients and CC patients showed comparable impairments in PF, except that the former group experienced greater impairment in the facet ‘*self‐esteem*’, which also relates to self‐appraisal and is part of the element ‘*identity*’, and less impairment in the facet ‘*impact of their behaviour on others*’ as part of the element *‘empathy’*. However, the group differences only reached small effect sizes. Second, when analysing the relationship between PF and indicators of AN severity, greater impairment in the domain of ‘*self‐functioning*’ and its underlying element ‘*identity*’ was moderately associated with higher AN severity as assessed by the EDE. Third, higher impairments in the facet ‘*understanding others’*, part of the ‘*empathy*’ element and in the facet ‘*connection*’ part of the ‘*intimacy*’ element were moderately associated with higher AN severity, as assessed by BMI percentiles.

Our finding on lower ‘*self‐esteem*’ in AN patients is in line with previous studies that identified an association between low self‐esteem and a range of mental health problems, with particularly strong associations reported for eating, mood and substance abuse disorders (Silverstone & Salsali, 2003) including AN (Fairburn et al., 2003; Kästner et al., 2019). Furthermore, a recent randomized controlled trial revealed that a self‐esteem treatment supplement positively affected both self‐esteem and changes in ED pathology (Biney et al., 2022). Previous research has also highlighted the importance of the self‐appraisal part of the ‘*self‐esteem*’ facet. Paterson et al. (2007) found that self‐competence, including self‐appraisal, self‐efficacy and self‐confidence, was important in predicting ED symptoms and mediated the relationship between eating pathology, problem solving, and avoidance. The authors suggest that AN patients' self‐appraisal may affect their coping with problems and stress, and argue that low self‐competence should be addressed in treatment.

Our finding of less impairment in one facet of the element ‘*empathy*’ (i.e., ‘*impact*’) in AN patients differs from previous research on empathy deficits in AN (Calderoni et al., 2013; Kerr‐Gaffney et al., 2019). However, our finding aligns with studies that used performance‐based assessments rather than self‐reports. For example, Sfärlea et al. (2018) found comparable performance between adolescents with AN, major depression or healthy controls in facial emotion recognition.

The small effect sizes of group differences between adolescents with AN and CC regarding impairment in PF are consistent with studies that found no differences in impairment in PF between adolescents with PD and CC, whereas healthy controls showed significantly lower impairment (Weekers et al., 2021). Similarly, no differences in impairment in PF were found in adults with mood and anxiety disorders (Doubková et al., 2022). These findings reflect previous evidence that there is a strong correlation between psychological disorders and personality problems.

The moderate association between higher impairment in the element ‘*identity*’ with higher AN severity (using the EDE) aligns with prior studies of the association between impairment in PF and ED severity in primarily adult clinical and community samples (Klein et al., 2022; Rohde et al., 2023). Additionally, a recent study has shown that impaired baseline identity is associated with lower weight gain during AN treatment, emphasising the importance of deficits in self‐functioning for treatment outcomes (Budde et al., 2022). These findings are congruent with contemporary theories, including those of Amianto et al. (2016) and Oldershaw et al. (2019), who propose that AN may evolve from self and identity deficits or a ‘lost sense of emotional self’ and indicate that impairment in PF may precede AN.

However, the opposite relationship between AN and impairment in PF must be considered, namely that AN may exacerbate existing impairments in PF. For example, patients suffering from AN typically tend to avoid unwanted emotions or experiences (Rawal et al., 2010). This may hinder their personal development and, thus, affect their PF, ultimately creating a vicious cycle of perpetuating factors. In line with this, ED psychopathology has been shown to be associated with impaired identity development, which is an integral part of PF (Verschueren et al., 2020).

A recent longitudinal study underscores the simultaneous dynamic interplay between identity and ED symptoms among adolescent and adult inpatients with various ED diagnoses, by finding an association between lower levels of drive for thinness or body dissatisfaction and a reduction in identity confusion and an increase in identity synthesis, thus emphasising the importance of identity development in treatment (Verschueren et al., 2024). This supports a possible bidirectional link between identity and AN psychopathology.

The moderate association between higher impairment in one facet of the element ‘*empathy*’ (i.e., ‘*understanding others*’) and higher AN severity (using BMI percentiles) is consistent with previous research on empathy deficits in AN (Calderoni et al., 2013; Kerr‐Gaffney et al., 2019). Interestingly, while lower BMI percentiles are associated with deficits in this specific aspect of empathy, our results also suggest that AN patients appear to be less impaired in another facet of empathy (*‘impact’*) compared to CC. This may suggest that empathy deficits in AN are more nuanced, with different facets of empathy affected to different degrees depending on BMI and the specific aspect of empathy.

The moderate association between higher impairment in one facet of the element ‘*intimacy*’ (i.e., ‘*connection*’) and higher AN severity (using BMI percentiles) is consistent with previous findings showing that recovered individuals with AN have greater motivation for intimacy than individuals currently suffering from full‐threshold AN (Brockmeyer et al., 2013). Both findings align with Treasure et al.’s (2020) cognitive‐interpersonal model, which describes a reinforcing cycle between social‐cognitive deficits in AN and interpersonal problems in close relationships that might constitute intimacy and empathy.

The lack of a significant correlation between impairment in PF and BMI *z*‐scores at admission compared to the EDE may be due to the characteristics of the EDE, which assesses cognitive‐emotional and behavioural symptoms of AN within the last 28 days and covers a broader range of different symptoms of AN than weight measures. This is supported by a study of adolescents with AN in which lower admission weight (BMI *z*‐scores) did not predict EDE score severity, but greater weight loss in the months prior to admission did predict EDE severity (Whitelaw et al., 2018). The results suggest that these indices of AN severity cover different aspects, and that impairment in PF may be related to cognitive and behavioural manifestations rather than with BMI *z*‐scores. However, a significant correlation was found between PF impairment and BMI percentiles, suggesting that BMI percentiles may better reflect physical severity in relation to functional impairment than BMI *z*‐scores.

### 4.1 Clinical implications

Examining impairments in PF in patients suffering from AN is essential to identify possible relevant factors that contribute to the development and maintenance of AN and that could represent treatment targets. Our results highlight the importance of addressing self‐functioning, particularly impaired self‐esteem (Biney et al., 2022), and identity development (Budde et al., 2022; Verschueren et al., 2024) in AN treatment. This is consistent with current psychotherapy manuals for AN (Goss & Allan, 2010; Schmidt et al., 2018), which already include the promotion of identity and self‐esteem, recognising their increased importance during adolescence, when these issues are naturally more pronounced due to developmental factors. Furthermore, it is imperative to address impairments in intimacy and empathy, that may occur in the development of AN, both with patients and their families. This approach is essential in order to disrupt the potentially perpetuating interpersonal dynamics that are associated with AN (Treasure et al., 2020).

### 4.2 Strengths and limitations

This is the first study that examined impairments in PF in adolescent inpatients suffering from AN based on the AMPD. We compared the results with a matched CC group, which allowed conclusions on specificity of findings for the diagnosis of AN. Another strength of this study is the application of validated semi‐structured interviews. However, certain limitations should be taken into account when interpreting our results. First, our analyses are cross‐sectional and no causal conclusions can be drawn. Second, all participants in our sample were female, which limits the generalisability of our findings. Third, our AN sample was rather small and the CC was heterogeneous (large variety of mental disorders). Fourth, we did not include a healthy control group, which would have contributed to the clarification of group differences in impairments in PF. However, given the strong association between mental disorders and PDs, we would expect to find very low impairment of PF in individuals without psychiatric diagnoses. Fifth, we only measured PF and AN severity at baseline and did not perform additional assessments during or at the end of the treatment that would allow us to draw conclusions about the influence of PF on treatment outcome. Future studies with larger sample sizes and a longitudinal design should investigate the association between impairments in PF and psychopathology, including ED pathology. It would be important to explore how impairment in PF affects AN development, severity and treatment outcomes and vice versa. In addition, considering maladaptive personality traits (criterion B) may offer additional insights into the intercorrelation between personality pathology and AN.

### 4.3 Conclusion

This is the first study to investigate impairment in PF using the STiP‐5.1 in adolescents with AN compared to a CC group and its relationship to the severity of AN. The results indicate that in terms of impairment in PF, patients with AN showed more deficits in self‐esteem than CC. Additionally, impairment in self‐functioning was found to be a moderate indicator of greater symptom severity in patients with AN. Higher impairment in interpersonal functioning was also found to be a moderate indicator of greater AN severity (lower BMI percentiles). Both findings suggest that self‐functioning and interpersonal functioning are potential treatment targets.

## CONFLICT OF INTEREST STATEMENT

The authors declare no conflicts of interest.

## Supporting information

Supporting Information S1

## Data Availability

The data that support the findings of this study are available from the corresponding author upon reasonable request.
