# Supplementary material for: Impairments in personality functioning in adolescents with anorexia nervosa
Source: Eur Eat Disord Rev. 2024 Oct 28;33(2):360–73. doi: 10.1002/erv.3146 (PMC11786945; doi:10.1002/erv.3146)
Supplement: Supplementary file 1 — Supporting Information S1 [file ERV-33-360-s001.docx]

**Supporting Information for**

***“Impairments in personality functioning in adolescents with anorexia nervosa.”***

Andrea M. Schumacher^1^, Armita Tschitsaz^1^, Stefan Lerch^1^, Andrea Wyssen^1^, Franziska Schlensog-Schuster^1^, Ines Mürner-Lavanchy^1,2^, Julian Koenig^3^, Marialuisa Cavelti^1^, Michael Kaess^1,4^

**Table of contents:** 6 eTables, standard MS Office format (Word)

- **eTable 1**. *Overview of core domains, elements and facets as measured by the Level of Personality Functioning Scale (Hutsebaut et al., 2014)*
- **eTable 2**. *Univariate analysis results for Pearson correlation between PF and AN severity*
- **eTable 3.**  *STiP-5.1 means and standard deviations in the total sample and by clinical status (AN and CC)*
- **eTable 4.** *Overall model fit of group differences in PF between AN patients and CC*
- **eTable 5.** *Overall model fit of multiple regression analyses in patients with AN: Prediction of AN severity by PF*
- **eTable 6.** *Group differences in PF between AN and CC patients (excluding patients with bulimia nervosa)*

**eTable 1**

*Overview of core domains, elements and facets as measured by the Level of Personality Functioning Scale (Hutsebaut et al., 2014)*

| Core domains | Elements | Facets |
| --- | --- | --- |
| Self-functioning | Identity | Experience of oneself as unique, with clear boundaries between self and others |
|  |  | Stability of self-esteem and accuracy of self-appraisal |
|  |  | Capacity for, and ability to regulate, a range of emotional experience |
|  | Self-direction | Pursuit of coherent and meaningful short-term and life goals |
|  |  | Utilization of constructive and prosocial internal standards of behaviour |
|  |  | Ability to self-direct productively |
| Interpersonal functioning | Empathy | Comprehension and appreciation of others’ experiences and motivations |
|  |  | Tolerance of differing perspectives |
|  |  | Understanding the effects of one’s own behaviour on others |
|  | Intimacy | Depth and duration of connection with others |
|  |  | Desire and capacity for closeness |
|  |  | Mutuality of regard as reflected in interpersonal behaviour |

**eTable 2**

*Univariate analysis results for Pearson correlation between PF and AN severity*

| Variable | EDE global | *p* | *p adj.* | BMI P† | *p*† | *p adj.* † | BMI *z* | *p* | *p adj.* |
| --- | --- | --- | --- | --- | --- | --- | --- | --- | --- |
| STiP-5.1 |  |  |  |  |  |  |  |  |  |
| **Total Score** | 0.33 | .030* | .029* | 0.02 | .925 | .930 | 0.02 | .910 | .911 |
| **Domain** |  |  |  |  |  |  |  |  |  |
| Self | 0.44 | .004** | .003** | 0.06 | .707 | .716 | 0.08 | .594 | .598 |
| Interpersonal | 0.14 | .360 | .358 | -0.04 | .808 | .804 | -0.06 | .697 | .699 |
| **Element** |  |  |  |  |  |  |  |  |  |
| Identity | 0.48 | .001** | .001*** | 0.03 | .852 | .852 | 0.14 | .373 | .368 |
| Self-direction | 0.33 | .031* | .029* | 0.08 | .606 | .614 | 0.02 | .914 | .916 |
| Empathy | 0.05 | .750 | .749 | 0.02 | .878 | .872 | -0.03 | .851 | .853 |
| Intimacy | 0.19 | .211 | .207 | -0.08 | .602 | .619 | -0.08 | .625 | .622 |
| **Facet**  Identity |  |  |  |  |  |  |  |  |  |
| Experience of oneself as unique | 0.41 | .006** | .004** | 0.08 | .608 | .623 | 0.05 | .769 | .768 |
| Self-esteem | 0.26 | .094 | .089 | 0.06 | .688 | .703 | 0.12 | .427 | .430 |
| Emotions | 0.49 | .001*** | .001*** | -0.07 | .676 | .682 | 0.17 | .277 | .271 |
| Self-direction  Goals | 0.22 | .156 | .155 | 0.23 | .142 | .144 | 0.13 | .420 | .420 |
| Norms | 0.16 | .291 | .288 | 0.11 | .495 | .499 | -0.05 | .760 | .757 |
| Self-reflection | 0.43 | .004** | .004** | -0.15 | .338 | .364 | -0.03 | .844 | .842 |
| Empathy  Understanding others | -0.05 | .743 | .751 | -0.17 | .283 | .278 | -0.10 | .528 | .541 |
| Perspectives | 0.10 | .525 | .523 | 0.21 | .191 | .184 | 0.01 | .925 | .926 |
| Impact | 0.08 | .613 | .619 | 0.05 | .769 | .786 | 0.02 | .892 | .898 |
| Intimacy  Connection | 0.28 | .066 | .063 | -0.14 | .373 | .395 | -0.03 | .864 | .868 |
| Closeness | 0.07 | .669 | .673 | -0.10 | .537 | .555 | -0.13 | .415 | .419 |
| Mutuality | 0.11 | .477 | .470 | 0.06 | .724 | .729 | -0.05 | .759 | .760 |

*Note.* n = 43; STiP-5.1 = Semi structured Interview for Personality functioning *Diagnostic and Statistical Manual of Mental Disorders, Fifth Edition;* EDE global = Eating Disorder Examination global score; BMI P = Body mass index percentiles; BMI *z* = Body mass index percentile *z*-scores; *p* adj. = adjusted *p*-value.

† BMI P values based on n = 42 as one outlier was removed

* *p* < .05 ** *p* < .01 *** *p* < .001

**eTable 3**

*STiP-5.1 means and standard deviations in the total sample and by clinical status (AN and CC)*

| STiP-5.1 scores (mean, *SD)* | Total sample (n = 170) | AN (n = 43) | CC (n = 127) |
| --- | --- | --- | --- |
| **Total score** | 1.29, 0.74 | 1.13, 0.73 | 1.34, 0.74 |
| **Domain** |  |  |  |
| Self | 1.70, 0.87 | 1.54, 0.86 | 1.75, 0.87 |
| Interpersonal | 0.88, 0.77 | 0.72, 0.75 | 0.93, 0.76 |
| **Element** |  |  |  |
| Identity | 1.94, 0.94 | 1.79, 0.92 | 1.99, 0.95 |
| Self-direction | 1.45, 0.96 | 1.29, 0.92 | 1.51, 0.97 |
| Empathy | 0.81, 0.81 | 0.56, 0.73 | 0.89, 0.82 |
| Intimacy | 0.95, 0.91 | 0.88, 0.92 | 0.97, 0.91 |
| **Facet** |  |  |  |
| Identity  Experience of oneself as unique | 1.54, 1.19 | 1.35, 1.17 | 1.61, 1.19 |
| Self-esteem | 2.23, 1.07 | 2.37, 1.02 | 2.18, 1.09 |
| Emotions  Self-direction | 2.04, 1.15 | 1.65, 1.19 | 2.17, 1.11 |
| Goals | 1.31, 1.20 | 1.09, 1.11 | 1.38, 1.22 |
| Norms† | 1.37, 1.32 | 1.33, 1.27 | 1.38, 1.34 |
| Self-reflection  Empathy | 1.69, 1.16 | 1.47, 1.05 | 1.76, 1.18 |
| Understanding others‡ | 0.64, 1.02 | 0.47, 0.98 | 0.71, 1.03 |
| Perspectives | 0.74, 0.94 | 0.53, 0.85 | 0.81, 0.97 |
| Impact  Intimacy | 1.04, 1.10 | 0.67, 0.94 | 1.17, 1.12 |
| Connection | 1.15, 1.19 | 1.07, 1.28 | 1.17, 1.16 |
| Closeness | 0.97, 1.10 | 0.93, 1.03 | 0.98, 1.13 |
| Mutuality | 0.73, 0.96 | 0.63, 0.95 | 0.76, 0.96 |

*Note.* AN = Anorexia nervosa group, CC = Clinical control group; STiP-5.1 = Semi-structured Interview for Personality Functioning *Diagnostic and Statistical Manual of Mental Disorders, Fifth Edition.*

†Two missing values in the total sample and CC group.

‡One missing value in the total sample and CC group.

**eTable 4**

*Overall model fit of group differences in PF between AN patients and CC*

| Model summary STiP-5.1  (n = 170) | *F*(3, 166) | *p* | *R^2^ adj.* | |
| --- | --- | --- | --- | --- |
| **Total score** | 19.88 | <.001*** | | 0.251 |
| **Domain** |  |  | |  |
| Self | 20.67 | <.001*** | | 0.259 |
| Interpersonal | 10.89 | <.001*** | | 0.149 |
| **Element** |  |  | |  |
| Identity | 23.74 | <.001*** | | 0.288 |
| Self-direction | 11.19 | <.001*** | | 0.153 |
| Empathy | 10.47 | <.001*** | | 0.144 |
| Intimacy | 7.43 | <.001*** | | 0.102 |
| **Facet** |  |  | |  |
| Identity  Experience of oneself as unique | 11.28 | <.001*** | | 0.154 |
| Self-esteem | 23.63 | <.001*** | | 0.287 |
| Emotions  Self-direction | 14.73 | <.001*** | | 0.196 |
| Goals | 8.00 | <.001*** | | 0.111 |
| Norms† | 4.82 | .003** | | 0.065 |
| Self-reflection  Empathy | 7.74 | <.001*** | | 0.107 |
| Understanding others† | 4.54 | .004** | | 0.063 |
| Perspectives | 7.98 | <.001*** | | 0.110 |
| Impact  Intimacy | 6.98 | <.001*** | | 0.096 |
| Connection | 3.09 | .029* | | 0.036 |
| Closeness | 7.49 | <.001*** | | 0.103 |
| Mutuality | 5.61 | .001** | | 0.076 |

*Note.* STiP-5.1 = Semi-structured Interview for personality functioning *Diagnostic and Statistical Manual of Mental Disorders, Fifth Edition; R^2^ adj*. = adjusted *R^2^* value.

†One missing value, n = 169, *F*(3, 165).

* *p* < .05 ** *p* < .01 *** *p* < .001

**eTable 5**

*Overall model fit of multiple regression analyses in patients with AN: Prediction of AN severity by PF*

| Model summary STiP-5.1 (n = 43) | *F*‡ | *p* | *R^2^ adj.* |
| --- | --- | --- | --- |
| EDE global†  Total score | 2.56 | .090 | 0.069 |
| Domain  Element  Facet | 3.65  2.57  1.39 | .021*  .043*  .223 | 0.159  0.158  0.108 |
| BMI P†§  Total score  Domain  Element  Facet | 1.14  0.92  0.87  2.33 | .330  .441  .514  .030* | 0.007  -0.006  -0.017  0.296 |
| BMI *z*†  Total score  Domain  Element  Facet | 0.87  0.95  1.11  0.81 | .427  .427  .370  .650 | -0.006  -0.004  0.013  -0.064 |

*Note.* STiP-5.1 = Semi structured Interview for Personality functioning *Diagnostic and Statistical Manual of Mental Disorders, Fifth Edition;* EDE global = Eating Disorder Examination global score; BMI P = Body mass index percentiles; BMI *z* = Body mass index percentile *z*-scores; *R^2^ adj*. = adjusted *R^2^* value.

†Outcome variables: indicator of AN severity.

‡Total score: *F*(2,40), Domain: *F*(3,39), Element: *F*(5,37), Facet: *F*(13,29).

§BMI P values based on n = 42 as one outlier was removed; Total score: *F*(2,39), Domain: *F*(3,38), Element: *F*(5,36), Facet: *F*(13,28).

* *p* < .05

**eTable 6**

*Group differences in PF between AN and CC patients (excluding patients with bulimia nervosa)*

| STiP-5.1 | Group differences *B* | *SE B* | 95% CI | *p* | *p adj*. | *f^2^* |
| --- | --- | --- | --- | --- | --- | --- |
|  |  |  | *LL UL* |  |  |  |
| **Total score** | -0.054 | 0.114 | -0.280 0.172 | .636 | .673 | 0.001 |
| **Domain**  Self | -0.024 | 0.139 | -0.298 0.250 | .863 | .876 | 0.000 |
| Interpersonal | -0.084 | 0.119 | -0.320 0.151 | .481 | .507 | 0.003 |
| **Element**  Identity | -0.006 | 0.146 | -0.295 0.283 | .968 | .968 | 0.000 |
| Self-direction | -0.042 | 0.163 | -0.365 0.281 | .797 | .822 | 0.000 |
| Empathy | -0.210 | 0.122 | -0.452 0.031 | .087 | .097 | 0.020 |
| Intimacy | 0.042 | 0.151 | -0.257 0.341 | .781 | .803 | 0.001 |
| **Facet**  Identity  Experience of oneself as unique | -0.106 | 0.200 | -0.502 0.290 | .599 | .639 | 0.002 |
| Self-esteem | 0.420 | 0.171 | 0.082 0.757 | .015* | .028* | 0.040 |
| Emotions  Self-direction | -0.332 | 0.188 | -0.704 0.041 | .080 | .112 | 0.021 |
| Goals | -0.095 | 0.207 | -0.503 0.314 | .648 | .682 | 0.001 |
| Norms† | 0.108 | 0.237 | -0.360 0.575 | .649 | .691 | 0.001 |
| Self-reflection  Empathy | -0.144 | 0.197 | -0.534 0.246 | .467 | .514 | 0.004 |
| Understanding others† | -0.100 | 0.166 | -0.428 0.227 | .546 | .558 | 0.002 |
| Perspectives | -0.140 | 0.148 | -0.433 0.153 | .347 | .366 | 0.006 |
| Impact  Intimacy | -0.394 | 0.172 | -0.733 -0.055 | .023* | .032* | 0.035 |
| Connection | 0.018 | 0.212 | -0.401 0.436 | .934 | .943 | 0.000 |
| Closeness | 0.078 | 0.185 | -0.288 0 .444 | .675 | .709 | 0.001 |
| Mutuality | 0.031 | 0.155 | -0.275 0.336 | .843 | .851 | 0.000 |

*Note.* n = 154; STiP-5.1 = Semi-structured Interview for personality functioning *Diagnostic and Statistical Manual of Mental Disorders, Fifth Edition; B =* unstandardized regression coefficient; *SE* *B* = standard error of *B*; LL = lower level; UL = upper level; *p* adj. = adjusted *p*-value;

*f^2^ =* Cohen’s *f^2^*.

†One missing value, n = 153.

* *p* < .05

**References**

Hutsebaut, J., Berghuis, H., De Saeger, H., Kaasenbrood, A., & Ingenhoven, T. (2014). *Semi-structured Interview for Personality Functioning DSM-5 (STiP-5)*. The Podium DSM-5 Research Group of the Netherlands Centre of Expertise on Personality Disorders. Utrecht, the Netherlands: Trimbos Institute.
